# Ethanol Vapor Sensing Properties of Triangular Silver Nanostructures Based on Localized Surface Plasmon Resonance

**DOI:** 10.3390/s110908643

**Published:** 2011-09-05

**Authors:** Wenying Ma, Huan Yang, Weimin Wang, Ping Gao, Jun Yao

**Affiliations:** 1 Department of Communication Engineering, Chengdu University of Information Technology, Chengdu 610225, China; 2 State Key Lab of Optical Technologies for Microfabrication, Institute of Optics and Electronics, Chinese Academy of Science, Chengdu 610209, China; E-Mails: yanghuan@ustc.edu (H.Y.); wwm06@mail.tsinghua.edu.cn (W.W.); pinggao1986@gmail.com (P.G.); junyao@ioe.ac.cn (J.Y.)

**Keywords:** localized surface plasmon resonance, volatile organic vapor, triangular nanoprism, sensitivity

## Abstract

A sensitive volatile organic vapor sensor based on the LSPR properties of silver triangular nanoprisms is proposed in this paper. The triangular nanoprisms were fabricated by a nanosphere lithography (NSL) method. They have sharp vertices and edges, and are arranged in an ideal hexangular array. These characteristics ensure that they exhibit an excellent LSPR spectrum and a high sensitivity to the exterior environment changes. The LSPR spectra responding to ethanol vapor and four other volatile organic vapors—acetone, benzene, hexane and propanol—were measured with a UV-vis spectrometer in real time. Compared with the other four vapors, ethanol exhibits the highest sensitivity (∼0.1 nm/mg L^−1^) and the lowest detection limit (∼10 mg/L) in the spectral tests. The ethanol vapor test process is also fast (∼4 s) and reversible. These insights demonstrate that the triangular nanoprism based nano-sensor can be used in ethanol vapor detection applications.

## 1. Introduction

In recent years, rapid, sensitive and well selective gas phase chemical sensors, especially sensors for detection of volatile organic vapors, have found significant applications in environmental monitoring, national security and food safety areas. Organic vapor sensors based on semi-conductive metal oxides (e.g., ZnO, SnO_2_) [1–4], surface acoustic waves (SAW) [5,6] and fiber waveguide optics [7,8] have been extensively studied in the past few decades. Each sensing technique possesses its own inherent advantages and limitations, and each sensor type has a specific role in its applicable field. Nevertheless, the gas sensing performance strongly depends on the surface-to-volume ratio (*i.e.*, specific area) of the materials for detection of volatile organic vapors, and nano-scale sensing materials or structures are expected to exhibit better sensing performance than bulk or thin film sensors [9].

Several research reports have confirmed the validity of this statement. Rella *et al*. demonstrated the good response to NO_2_ and CO when the size of SnO_2_ grain was controlled below 10 nm [10]. Wan and coworkers found that the gas sensors based on ZnO nanowires fabricated with micro-electro-mechanical system (MEMS) technology exhibited high sensitivity and fast response times to ethanol gas at a working temperature of 300 °C [11]. Although nano-scale metal oxide grain significantly enhanced the sensing performance of the correlated sensor, the working temperature (150∼600 °C), required for maximum response prevents their practical applications in most areas.

Besides research on nanopowders as the assistant material to enhance the sensor response, studies of nanostructures themselves working as both receptor material and transducer are also coming to researchers’ attention. Noble metal nanostructures are one type of interesting nanostructures due to their significant potentials in optical communications [12,13], sub-wavelength lithography [14], and bio-chemical sensing applications [15,16]. Most of those applications are based on the unique optical properties of Localized Surface Plasmon Resonance (LSPR). LSPR occurs when the frequency of the incident light is resonant with the collective oscillations of the conduction electrons in the metal nanoparticles (MNPs), and externally expressed by a strong extinction (the sum of absorption and scattering) band in the visible spectrum. The location of the extinction maximum is highly dependent on the dielectric properties of the surrounding environment. As a result, the peak wavelength shifts in the extinction maximum of nanoparticles can be used to detect molecule-induced changes surrounding the nanoparticles.

Recently, several papers have been published on the use of the LSPR spectrum technique for the detection of streptavidin [17], anti-biotin [18], concanavalin [19], Alzheimer disease bio-markers [20], and other bio-recognition events [21]. There are also reports on LSPR sensing applications in the liquid phase for the detection of organophosphorous pesticides [22], hydrogen peroxide [23], ammonia [24], and other chemical detection experiments. In terms of gas phase molecule detection, besides several studies in inorganic gases sensing [25–27], experiments by Chen *et al*. [28,29] were carried out to explore the LSPR spectra response to a series of organic gases such as octane, pentanol, *etc.* by utilizing silver nanoparticles and gold nanoshells. Vaskevich and coworkers [30] found that a polymer-coated gold island showed selective gas sensing characteristics by investigating the extinction spectrum response to several vapors qualitatively, without obtaining the exact sensing sensitivity and detection limits.

In this paper, a sensitive organic vapor sensor based on silver triangular nanoprisms array was developed. Its sensing properties for ethanol relative to other four vapors (acetone, benzene, propanol and hexane) were investigated. The silver triangular nanostructures with sharp vertices and edges, proved to be more sensitive than the traditional spherical nanoparticles [31,32], and were prepared by a nanosphere lithography (NSL) fabrication method. The LSPR spectra response to five organic vapors was measured by a UV-vis spectrometer in real time. Besides, the sensing sensitivity and detection limit of five organic vapors, the linearity of spectra changes, the time response, as well as the repeatability were also discussed and analyzed in detail.

## 2. Experimental Setup and Method

### 2.1. Substrate

K9 glass substrates (20 mm × 10 mm × 1.5 mm) were used to provide a good light transmissivity and a physical support for the triangular nanoprisms. To ensure hydrophilicity and cleanness, the substrates were prepared as follows: first, immersed in a piranha solution (30% H_2_O_2_/H_2_SO_4_ = 1/3) at 80 °C for 30 min; second, after cooled in N_2_ and rinsed with copious amounts of double distilled water, the substrates were sonicated in 5:1:1 H_2_O/NH_3_·H_2_O/30%H_2_O_2_; third, rinsed repeatedly with de-ionized water and stored in it before nanostructures fabrication process.

### 2.2. Transducer Fabrication

As a transducer of light signals and vapor concentration, silver triangular nanoprisms present a good extinction peak and a large surface area to facilitate quantitative adsorption of organic vapors. The NSL technique, one of the most popular means to fabricate large area periodic nanostructure arrays, was employed to create the triangular nanoprisms in the experiment due to its time and money cost effectiveness. The fabrication process primarily consists of three steps: first, a layer of ∼399 nm polystyrene (PS) nanosphere colloidal suspension (1.8%, 3400A, Thermo Scientific Inc., Canada) was spin coated (speed: 3,000 rpm) onto the substrate to form a large-area close-packed nanosphere monolayer pattern; second, a layer of ∼50 nm silver was deposited on the monolayer template in a thermal evaporator (400-I, C-Vac Inc., China); and third, the PS nanospheres were removed by dissolving them in ethanol with the aid of sonication for 20 s∼60 s. Following that, a well ordered two-dimensional triangular nanoprisms array was finally obtained on the substrate.

### 2.3. Detection System

**Figure 1.**
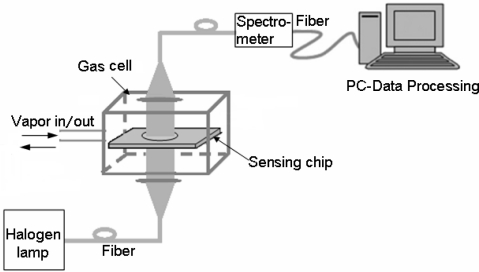
Schematic presentation of experimental setup.

The schematic diagram of the detection system is shown in Figure 1. A white light beam radiated by a halogen lamp was propagated through a multimode optical fiber to illuminate the sensing chip. On the other side of the chip, it was collected by another multimode fiber. The LSPR spectra were measured at room temperature (20 °C) by using a Scientific-grade UV-Vis Spectrometer (QE65000, Ocean Optics Inc., USA), with associated data processing software (SpectraSuite, Ocean Optics Inc.). The spectrometer was operated at a wavelength resolution of 1 nm in our experiment, and the extinction can be evaluated by:

(1) $$E(\lambda )=-{\text{log}}_{10}\left(\frac{S(\lambda )-D(\lambda )}{R(\lambda )-D(\lambda )}\right)$$

where S(λ), D(λ) and R(λ) denote the sample intensity, the dark intensity, and the reference intensity at the wavelength of λ, respectively. An airproof gas cell was used in this experiment to provide a platform where the test vapor was adsorbed on the transducer and the vapor concentration was transmitted into the optical signal. The gas cell was made of a specific kind of polymer and specially designed and fabricated to enhance the light transmission.

The test vapor of a defined concentration was prepared by injecting a specific volume of saturated vapor into the gas cell, where the saturated vapor was produced by stewing a certain amount of the corresponding matter of liquid phase in a big sealed container for several hours. The injection volume in experiment was defined as:

(2) $$V_{i}=\frac{C_{d}}{C_{s}}•V_{r}$$

where *C_d_*, *C_s_* and *V_r_* refer to the test vapor concentration, the saturated vapor concentration and the volume of gas cell, respectively.

## 3. Results and Discussion

**Figure 2.**
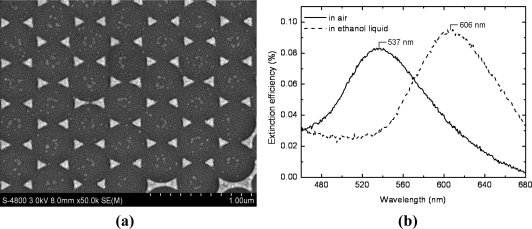
**(a)** SEM image of the fabricated triangular nanoprisms. **(b)** The test LSPR spectra in clean air and in liquid phase ethanol.

Figure 2 shows the SEM image and the LSPR spectrum of the fabricated triangular nanoprisms. As shown in Figure 2(a), the triangular nanosprisms with sharp vertices and edges were arrayed in a hexagonal form, with a period of 399 nm. This regular pattern gives an excellent LSPR spectrum in air, with a high peak to valley (PV) value, a narrow bandwidth, and an appropriate spectrum peak wavelength, 537 nm in air, as shown in Figure 2(b). Due to these characteristics, triangular nano-sensor has great advantages in detection sensitivity for use in gas phase chemical sensing.

### 3.1. LSPR Spectrum Response to Ethanol Vapor Concentrations

**Figure 3.**
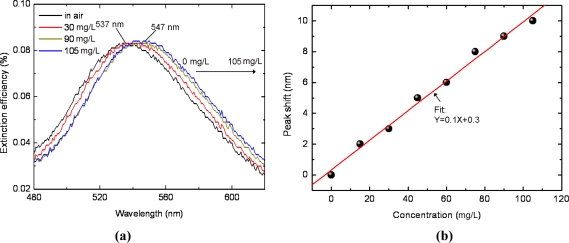
**(a)** LSPR spectra response to different ethanol vapor concentrations. **(b)** Response sensitivity of LSPR spectrum calibrated with eight different ethanol vapor concentrations.

According to the test results for the sensor chip exposed to ethanol vapor of different concentrations presented in Figure 3(a), the peak wavelength of the spectrum shifts from 537 nm to 547 nm gradually when the ethanol concentration varies from 0 mg/L (in air) to 105 mg/L. Comparing Figure 2(b) with Figure 3, it is apparent that the variation of peak wavelength for ethanol in gas phase is much smaller than that in liquid phase, indicating a much smaller refractive index of ethanol in gas phase than in liquid phase. This phenomenon is an interior interpretation of the difficulty of gas molecule detection for those refractive index based sensors.

In order to better resolve the overall relationship between the resonant wavelength and the ethanol vapor concentration, we performed more experiments on different vapor concentrations. The extinction spectra peak wavelength shift (0 mg/L as the base point) as a function of ethanol vapor concentration isplotted in Figure 3(b). As the concentration increases from 0 mg/L to 105 mg/L, in increment of 15 mg/L, the peak shift amount increases gradually, suggesting a linear relationship. Hence, we define “vapor sensor sensitivity” *m* to directly evaluate the sensing performance of metal nanostructures based volatile organic vapor sensors:

(3) $$m=\frac{{\Delta \lambda }_{p}(\text{nm})}{{\Delta C}_{d}(\text{mg}/L)}$$

where Δ*λ_p_* and Δ*C_d_* denote the peak wavelength shift in a certain vapor concentration relative to that in air and the corresponding concentration change. For the ethanol vapor test, the sensitivity is approximately 0.1 nm/mg L^−1^, as shown in Figure 3(b), indicating a 1 mg/L increment in ethanol vapor concentration will induce a 0.1 nm red shift in spectrum.

The detection limit, determined by the sensor sensitivity as well as the operational resolution of the applied detector, is approximately 10 mg/L for ethanol test in our experiment based on the results in Figure 3(b). It will be improved if a spectrometer with greater wavelength resolution is used.

### 3.2. Response Time and Repeatability

The reversibility, stability, and response time of the LSPR spectra were examined by repetitive intermittent exposure of the silver triangular nano-transducers to ethanol vapor. The saturated ethanol vapor, approximately 105 mg/L in room temperature, was switched on for 15 s and then switched off for 120 s by closing or opening the riser of the gas cell during the three test cycles. A response of the LSPR spectra was then observed upon the repetitive variation of the environment in the gas cell between clean air and saturated ethanol vapor-containing air.

**Figure 4.**
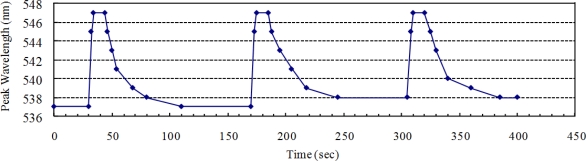
Time responds to saturated ethanol vapor of the nanosensor.

Figure 4 shows the dynamic peak wavelength shift changes as time increases in the three test cycles. After the 105 mg/L ethanol vapor was injected into the gas cell, a considerable peak wavelength response (10 nm) can be observed. This is quantitatively consistent with the experiment results in Section 3.1. As indicated in Figure 4, the peak wavelength response was rapid, stable, and reproducible. It should be noted that the recovery time (∼66 s for the first cycle) lasts much longer than the response time (∼4 s for the first cycle). This can be attributed to the difference of mixing time for ethanol molecule: when switched on, the concentration in the tiny closed gas cell grows from 0 mg/L to saturated in a short time; when switched off, the saturated ethanol vapor diffused into the exterior environment with a concentration in gas cell growing from saturated to approximately 0 mg/L in a much longer time.

It also should be noted that the peak wavelengths after the second test cycle and the third test cycle are a little bigger than that before and after the first test cycle. This is because the concentration of ethanol vapor in the gas cell cannot return to 0 mg/L (clean air) after two test cycles. The reversibility will be further improved when the test gas cell is created of vacuum after each test cycle.

### 3.3. Selectivity

**Figure 5.**
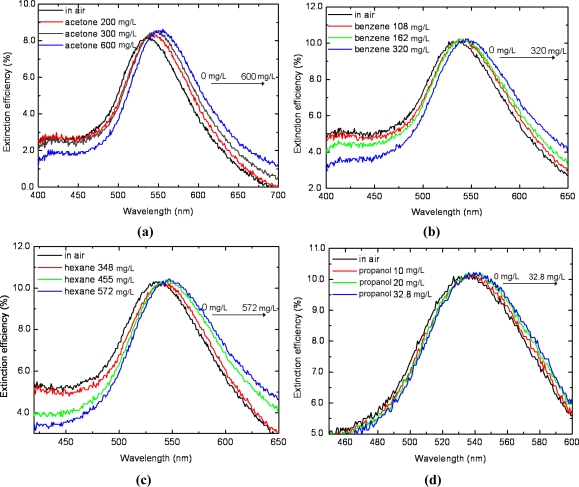
LSPR spectra responding to various vapors. **(a)** Acetone; **(b)** Benzene; **(c)** Hexane; **(d)** Propanol.

The LSPR spectrum response of silver triangular nanoprisms to other four analyte volatile organic vapors (*i.e.*, acetone, benzene, propanol and hexane) was tested. The sensor chip applied in this section was the one that applied in ethanol vapor test in Sections 3.1 and 3.2 for comparison. Figure 5 describes the LSPR spectra measured *in situ* during the sensor’s exposure to the four analyte vapors of various concentrations. The peak wavelengths vary as the concentration increases for the four test vapors, indicating different increments in refractive index in the gas cell. As indicated in Figure 5(a), the saturated acetone vapor induces a red shift of the LSPR spectrum of approximately 17 nm, much larger than that for propanol, 3 nm as shown in Figure 5(d). Also, both the maximum red shift for benzene vapor and hexane vapor is 10 nm, the same with ethanol vapor shown in Figure 3(a).

**Figure 6.**
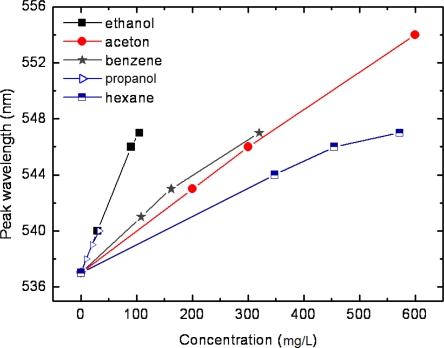
Calibration sensitivity curves of peak wavelength response to concentrations of five test vapors.

In order to better resolve the selective characteristics of the nano-sensor, we further made a comparison of the peak wavelength response to the concentration of the five test vapors, as shown in Figure 6. All the five test vapors, ethanol, acetone, benzene, propanol and hexane, showed a good relative linear response, with the slope of ethanol being the greatest. This reveals selectivity for ethanol of the LSPR sensor, and indicates the physical interaction between nanostructures and vapor molecules. On the one hand, as is well known that the LSPR spectrum responds only to the ambient refractive index change, it is more sensitive with compounds of a higher refractive index than that of a lower refractive index. On the other hand, changes in refractive index induced by compounds in gas phase are relatively smaller than that in liquid phase. This indicates that vapors with poor volatility (*i.e.*, low saturated vapor pressure), which are easy to liquefy on the surface of metal nanostructures, will exhibit a good LSPR sensitivity.

As described in Table 1, both the refractive index and saturated pressure contribute to the sensor response. Ethanol, with a relatively higher liquid refractive index and a lower saturated vapor pressure, exhibits a higher sensitivity than four other vapors. The detection limit of the ethanol is approximately 10 mg/L, much lower than that of the other four test vapors. Thus, the fabricated nano-transducer has potential applications in detection of ethanol in environmental monitoring and food safety.

## 4. Conclusions

The volatile organic vapor sensing properties of an LSPR-based triangular nanoprisms sensor were studied in this paper. The high surface area and the regular pattern of triangular nanostructures fabricated by NSL provide an effective method for vapor adsorption and a feasible way for high sensitive vapor detection. A red shift extinction spectrum can be observed as the concentration of the test vapors increases. The sensitivity, defined as the peak wavelength shift per vapor concentration change in ambient conditions, is approximately 0.1 nm/mg L^−1^ for ethanol This result, much higher than that for other four test vapors (propanol, benzene, acetone, and hexane) indicates a good selectivity for ethanol vapor by the sensor chip. The detection limit, determined by the sensitivity of the sensor and the wavelength resolution of the applied spectrometer, is approximately 10 mg/L for ethanol in our experiments. Besides, the triangular nanoprisms based ethanol vapor sensor has a relatively fast response time (∼4 s) and a good sensing stability and reversibility. This study indicates the great potential in practical applications in detection of ethanol vapors for the LSPR sensor.

## Figures and Tables

**Table 1.** Comparison of the physical characteristics and LSPR selectivity of the five test vapors.

**Volatile organic vapors**	**ethanol**	**propanol**	**benzene**	**acetone**	**hexane**
Refractive index (in liquid phase)	1.37	1.36	1.49	1.36	1.34
Saturated vapor pressure (mmHg)	40	10	75	185	121
sensitivity (nm/mg L^−1^)	0.1	0.095	0.031	0.028	0.018
Detection limit (mg/L)	10	10.5	32.3	35.7	55.5
